# Decidualization Process Induces Maternal Monocytes to Tolerogenic IL-10-Producing Dendritic Cells (DC-10)

**DOI:** 10.3389/fimmu.2020.01571

**Published:** 2020-08-18

**Authors:** Soledad Gori, Elizabeth Soczewski, Laura Fernández, Esteban Grasso, Lucila Gallino, Fatima Merech, Ana Colado, Mercedes Borge, Claudia Pérez Leirós, Gabriela Salamone, Rosanna Ramhorst

**Affiliations:** ^1^CONICET, Universidad de Buenos Aires, Instituto de Química Biológica de la Facultad de Ciencias Exactas y Naturales (IQUIBICEN), Buenos Aires, Argentina; ^2^Instituto de Medicina Experimental (IMEX)-CONICET, Academia Nacional de Medicina, Buenos Aires, Argentina

**Keywords:** decidualization, DC-10, dendritic cells, immunomodulation, HLA-G, myeloid regulatory cells

## Abstract

Decidualization is a process that involves phenotypic and functional changes of endometrial stromal cells to sustain endometrial receptivity and the participation of immunoregulatory factors to maintain immune homeostasis. In this context, tolerogenic dendritic cells (DCs) can induce regulatory T cells, which are essential to manage the pro- to anti-inflammatory transition during embryo implantation. Recently, Myeloid Regulatory Cells (MRCs) were proposed as immunosuppressants and tolerance-inducer cells, including the DC-10 subset. This novel and distinctive subset has the ability to produce IL-10 and to induce type 1 regulatory T cells (Tr1) through an HLA-G pathway. Here we focus on the impact of the decidualization process in conditioning peripheral monocytes to MRCs and the DC-10 subset, and their ability to induce regulatory T cells. An *in vitro* model of decidualization with the human endometrial stromal cell line (HESC), decidualized by medroxyprogesterone and dibutyryl-cAMP was used. Monocytes isolated from peripheral blood mononuclear cells from healthy women were cultured with rhGM-CSF + rhIL-4 and then, the effect of conditioned media from decidualized (Dec-CM) and non-decidualized cells (Non-dec-CM) was tested on monocyte cultures. We found that Dec-CM inhibited the differentiation to the CD1a^+^CD14^–^ immature DC profile in a concentration-dependent manner. Dec-CM also significantly increased the frequency of CD83^+^CD86^low^ and HLA-DR^+^ cells in the monocyte-derived culture. These markers, associated with the increased production of IL-10, are consistent with a MRCs tolerogenic profile. Interestingly, Dec-CM treatment displayed a higher expression of the characteristic markers of the tolerogenic DC-10 subset, HLA-G and ILT2/CD85j; while this modulation was not observed in cultures treated with Non-dec-CM. Moreover, when monocyte cultures with Dec-CM were challenged with LPS, they sustained a higher IL-10 production and prevented the increase of CD83, CD86, IL-12p70, and TNF-α expression. Finally, the DC-10 subset was able to induce a CD4^+^HLA-G^+^ regulatory T cells subset. These results suggest that the decidualization process might induce different subsets of MRCs, like DC-10, able to induce regulatory T cells as a novel CD4^+^HLA-G^+^ subset which might play an immunoregulatory role in embryo implantation.

## Introduction

The maternal immune system was subjected to opposing selective pressures over millions of years of evolution: on the one hand it recognizes microbial pathogens and responds to eliminate them, whereas on the other hand, it accepts semi-allogeneic fetuses without ignoring its existence. Hence, the embryo has an “immunoprivileged status” that allows the establishment of early pregnancy by instructing immune tolerance induction in the maternal immune system. Therefore, the feto-maternal interface is characterized by dynamism: the microenvironment changes as pregnancy progresses accompanied by immunological phases with different profiles (1). The changes of the maternal immune profile are strictly controlled by complex regulatory mechanisms at decidualization, implantation, and placentation.

Particularly, the decidualization program involves phenotypic and functional changes of endometrial stromal cells and not only sustains the endometrial receptivity, but also allows the secretion of immunoregulatory factors which may condition maternal leukocytes to a regulatory profile (2). This process is unique and characteristic of endometrium and, in humans, it is activated independently of the presence of the blastocyst (2, 3). In this context, even though myeloid dendritic cells (DCs) are only 1–2% of decidual leukocytes, they initiate the adaptive immunity and, therefore, they are crucial for the establishment of immunological tolerance (1, 4). DCs in human decidua represent a complex population and their number fluctuates through different phases of the menstrual cycle and during pregnancy (5–8). Interestingly, in the last few years, Myeloid Regulatory Cells (MRCs) have been proposed as immune-suppressors and tolerance-inducers including the DC-10 (9, 10). This novel subset represents tolerogenic DCs (Tol-DCs) which notably spontaneously secrete large amounts of IL-10 and express different tolerogenic markers such as membrane-HLA-G and its receptors immunoglobulin-like transcript (ILT) 2, ILT-3, and ILT-4 (11). In fact, DC-10 are able to induce T cells anergy and type 1 regulatory T cells (Tr1) through the IL-10-dependent ILT4/HLA-G pathway *in vitro* (11). Remarkably, even though a single stimulation of allogeneic naïve T cells with DC-10 is sufficient to generate allo-specific Tr1 cells, the chronicity of allogeneic stimulation reinforces Tr1 induction (12). Previous reports indicate a higher percentage of DC-10 into the human decidua compared to peripheral blood during the first trimester of pregnancy; but, it is still unknown if these cells are recruited to the decidua or induced *in situ* (6). Tol-DCs also have the ability to induce regulatory T cells (Tregs, CD4^+^FOXP3^+^), a critical role in pregnancy that was proven using several *in vivo* and *in vitro* approaches in murine models as well as in humans (13–17). Recently, T cell subsets, which do not express FOXP3, with immunosuppressive ability based on the increase in HLA-G expression and IL-10 production were reported (8). The expression of HLA-G on T cells could be induced by DCs (6, 18, 19). The frequency of CD4^+^HLA-G^+^ cells in peripheral blood increases in healthy pregnant women, being even more pronounced within the decidua (6, 18); however, it is still unclear whether the decidualization program modulates their induction.

Since T cells and DCs are critical to sustain homeostasis in pregnancy, here we focused on the impact of the decidualization process in conditioning peripheral monocytes to MRCs and, particularly to the DC-10 subset. Finally, we investigated the ability of DC-10 to induce different regulatory T cell subsets.

## Materials and Methods

### Reagents

Endotoxin-free reagents and plastic materials were used in all experiments. RPMI-1640, phosphate-buffered saline (PBS), Dulbecco’s modified Eagle’s medium (DMEM), fetal bovine serum (FBS), and penicillin/streptomycin were purchased from Gibco (Invitrogen, Argentina). Twenty-four-well flat bottom polystyrene plates were purchased from Jet-biofil (AP Biotech, Buenos Aires, Argentina) while 96-well U-bottom plates and half-area 96-well ELISA were obtained from Greiner Bio One (GBO, Buenos Aires, Argentina). Ficoll-Paque PLUS and Percoll were obtained from GE Healthcare Life Sciences (Embiotec, Buenos Aires, Argentina). Recombinant human IL-4 and recombinant human granulocyte-macrophage colony-stimulating factor (GM-CSF) were obtained from Miltenyi Biotec (Lab Systems, Buenos Aires, Argentina). Lipopolysaccharide (LPS) from *Escherichia coli* was purchased from Sigma-Aldrich (Merck, Argentina).

### Blood Samples

Buffy coats were obtained from fertile female volunteers, defined as women who had two or more previous normal pregnancies without any miscarriage in their clinical history, were non-smokers, and who were not under pharmacological treatment for at least 10 days before the day of sampling. The Investigation and Ethics Committees of ‘Academia Nacional de Medicina’ from CABA, Argentina have approved this study. All research was performed in accordance with relevant guidelines and regulations, and written informed consent for the collection of samples and subsequent analyses was obtained from all blood donors recruited by “Fundación Hemocentro Buenos Aires,” CABA, Argentina in accordance with the Declaration of Helsinki.

### Human Endometrial Stromal Cell Line Culture

The human endometrial stromal cell (HESC) line was maintained in DMEM-F12 supplemented with 10% FBS, 50 U/ml penicillin, 50 μg/ml streptomycin, and 2 mM glutamine (20, 21) (complete medium). This cell line was kindly provided by Dr. Gil Mor of Medical School, Yale University, United States.

*Decidualization*: HESC cells were cultured in 24-well plates until they reached 70% confluence with complete medium. Then, they were treated with medroxyprogesterone (MPA) (10^–7^M) and dibutyryl cAMP (db-cAMP) (2.5 × 10^–3^M) for 8 days (Dec), changing half of the culture media and renewing the stimuli every 48 h. The decidualization process was confirmed by the evaluation of decidual markers and cell viability, as previously described (22). Non-decidualized (Non-dec) cells were cultured simultaneously in similar conditions in absence of decidualization stimuli.

After 8 days of culture, Non-dec and Dec-HESC cells were washed three times and cultured in RPMI 1640 medium supplemented with 10% of heat inactivated FBS, 50 U/ml penicillin, and 50 μg/ml streptomycin (DC complete medium) for an additional 48 h and Conditioned Media (CM) were collected.

### Dendritic Cells Differentiation

Peripheral blood mononuclear cells (PBMC) were isolated from buffy coats by Ficoll-Paque PLUS density gradient centrifugation (1.077 g/mL). Monocytes were isolated by centrifugation on a discontinuous Percoll gradient with modifications of a previously described method (23, 24). Briefly, PBMC were suspended in Ca2^+^, Mg2^+^-free Tyrode’s solution supplemented with 0.2% EDTA and incubated for 45 min at 37°C. During this incubation, the osmolarity of the medium was gradually increased from 290 to 360 osmol/l by addition of NaCl. Two different Percoll fractions were layered in polypropylene tubes: 50% at the bottom followed by 40%. PBMC (40 × 10^6^/ml) were layered at the top and they were centrifuged at 620 g for 50 min at 4°C. Monocytes were recovered at the interface, washed, and the purity and viability were checked by flow cytometry analysis and trypan blue exclusion, respectively. The purity and the viability accepted in all cases were >85% and >95%, respectively.

To obtain immature DC (Media-treated cells), monocytes (1 × 10^6^/ml) were cultured in DC complete medium with 30 ng/ml IL-4 and 30 ng/ml GM-CSF in 96-well U-bottom plates for at least 5 days. The expression of CD1a/CD14 was measured to confirm the differentiation to immature DC as previously described (25). In parallel, monocytes were also cultured in DC complete medium with IL-4 + GM-CSF in presence of HESC-CM (Non-dec-CM or Dec-CM). On the last day, cell supernatants were collected, and the phenotype was analyzed by flow cytometry. In some cases, on day 5, the cells were treated with LPS 0.2 μg/ml for 18 h if was required for the assays.

All experiments were performed independently using different donor monocytes (N is indicated in the legend of each figure).

### Endocytosis Assay of FITC-OVA

At day 6 of differentiation, monocyte-derived cells were suspended at 2 × 10^6^ cells/ml in fresh medium. FITC-Ovalbumin (FITC-OVA) was added at a final concentration of 100 μg/ml and cells were incubated for 25 min at 37°C. In parallel, a control was incubated on ice to determine unspecific binding. Cells were washed two times with ice-cold 2% FBS/PBS and fixed with 1% paraformaldehyde. The FITC-OVA uptake was then evaluated by flow cytometry as we have previously described (26).

### Mixed Lymphocyte Reaction

Monocytes (5 × 10^4^ cells/100 μl) were differentiated in presence or absence of 1:2 dilution HESC-CM for 6 days. The obtained monocyte-derived cells were then suspended in DC complete medium with 2.5 × 10^5^ freshly isolated allogeneic lymphocytes (DC/lymphocyte ratio = 1/5) and cultured for 5 days more as we have previously described (25). The monocytes and lymphocytes used for mixed lymphocyte reaction (MLR) were isolated by centrifugation on a discontinuous Percoll gradient described above, reaching a purity >90 and >95%, respectively. At the last day of MLR, we evaluated the expression of different markers on T cells by flow cytometry and their cytokine production profile was evaluated in cell supernatants by ELISA.

### Flow Cytometry

Cells were washed with PBS supplemented with 2% FBS/PBS and FITC-, APC- and PE-conjugated mAbs directed to CD1a, CD14, CD86, HLA-DR, CD83, CD4, CD25 (BD Biosciences), ILT-2/CD85j, and HLA-G (BioLegend, San Diego, CA, United States) or the corresponding isotype controls were added at saturating concentrations for 30 min at 4°C. Then, two additional washes were performed, and cells were fixed with 1% paraformaldehyde. Stained cells were acquired using an FACS Calibur and FACSAria II cytometers and results were analyzed using FlowJo 7.6 Software.

### Measurement of Cytokines by ELISA

Cytokines were evaluated in cell supernatants using commercial kits: IL-10, IL-12p70, TNF-α, and IFN-γ (BD Biosciences), according to the manufacturer’s recommendations.

### Statistical Analysis

GraphPad Prism (GraphPad Software Inc., San Diego, CA, United States) was used to perform all statistical tests. Statistical significance was determined using the non-parametric Friedman test with Dunn’s multiple comparisons post-test. Statistical significance was defined as *p* < 0.05 and exact *p*-values and comparisons were indicated in each graph.

## Results

### Decidualized Cells Inhibit Monocyte Differentiation to CD1a^+^CD14^–^ Immature DC Profile in a Concentration-Dependent Manner

**FIGURE 1:**
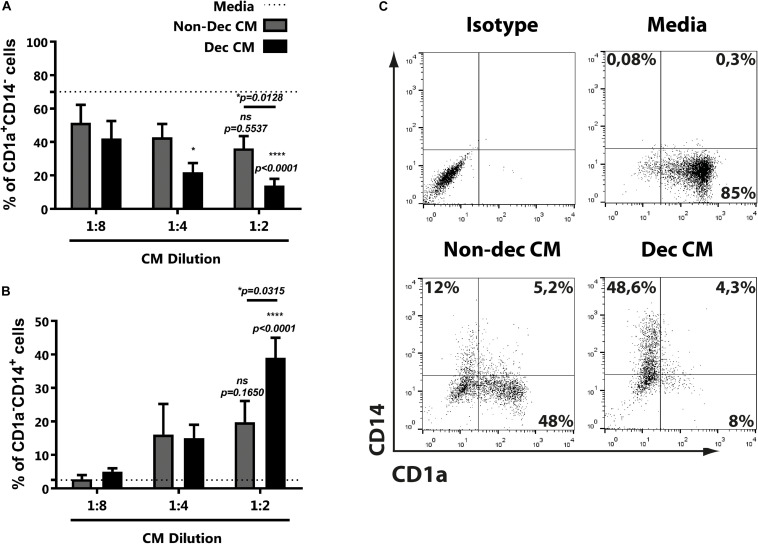
Decidualized cells inhibit monocyte differentiation to immature DC in a concentration-dependent manner. Monocytes were cultured to differentiate to immature DC in absence (Media) or presence of different dilutions of Non-dec or Dec-CM for 5 days. Then, the CD1a/CD14 expression was evaluated by flow cytometry. **(A,B)** Mean ± SEM of positive cells from at least eight experiments are shown. Dotted lines represent the mean of positive cells of Media-treated culture. **(C)** Representative experiment of 1:2 dilution of CM is shown. The statistical test used is the Friedman test with Dunn’s multiple comparisons post-test. *p*-values and comparisons were indicated in graph; *p*-values without lines indicate comparisons with dotted lines (Media).

Considering that endometrial stromal cells change their secretome during the decidualization process, including the production of immunoregulators, we evaluated the influence of conditioned media (CM) of decidualized (Dec) and non-decidualized (Non-dec) HESC cells on immature DC differentiation. Monocytes were cultured to differentiate into immature DC with GM-CSF + IL-4 in absence (Media) or presence of different dilutions of Non-dec or Dec-CM for 5 days. As Figure 1A shows, Dec-CM inhibited monocyte differentiation to CD1a^+^CD14^–^ immature DC profile in a concentration-dependent manner. On the other hand, this effect was also accompanied by a persistence of CD1a^–^CD14^+^ cells (Figure 1B). Figure 1C shows representative dotplots of the immunostaining of DC differentiated in the absence or presence of CM from endometrial cells before and after decidualization.

Altogether, the present results suggest that CM from endometrial cells, after decidualization, interfere with DC differentiation while it increases the frequency of CD1a^–^CD14^+^.

### Decidualized Cells Induce a Myeloid Regulatory Cells-Profile on Monocyte-Derived Cultures

**FIGURE 2:**
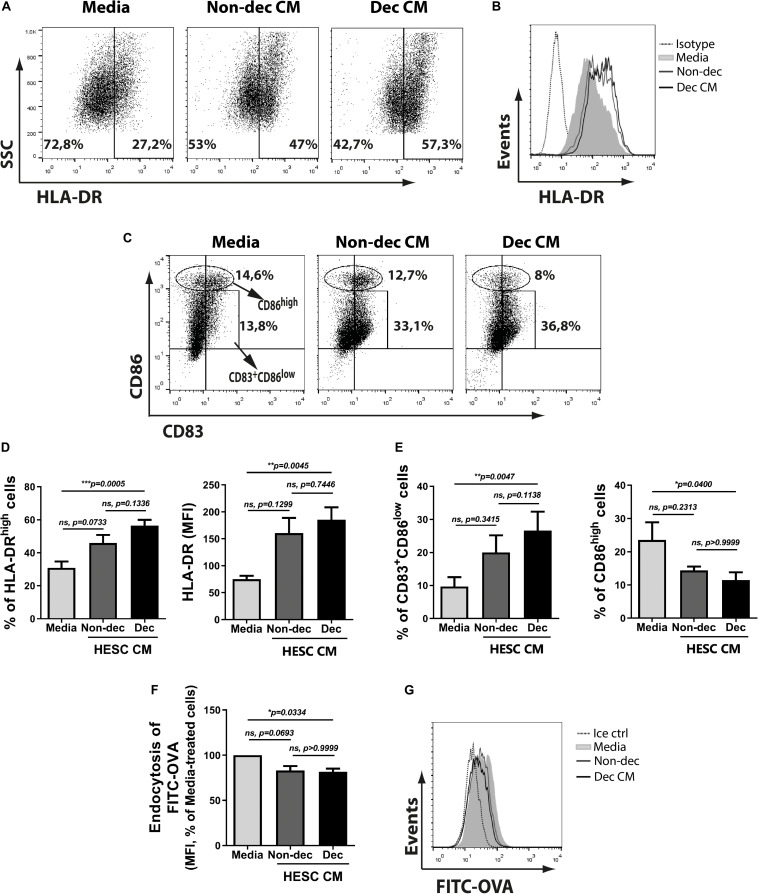
Decidualized cells induce an MRC-profile on monocyte-derived cultures with higher frequency of CD83^+^CD86^low^ and HLA-DR^high^ cells. Monocytes were cultured to differentiate to immature DC in absence (Media) or presence of 1:2 dilution of Non-dec or Dec-CM for 5 days. **(A–E)** After differentiation, the expression of HLA-DR, CD86, and CD83 was measured by flow cytometry. Representative experiments are shown in panels **(A–C)** and the mean ± SEM of positive cells or MFI from five to eight experiments is shown in panels **(D,E)**. **(F,G)** On day 6, cells were washed and stimulated with OVA-FITC in fresh medium for 25 min at 37°C and the endocytic ability was evaluated by flow cytometry. Cells incubated with FITC-OVA in ice were used as negative control. The mean ± SEM of MFI from seven experiments is shown in panel **(F)** and the representative experiment is shown in panel **(G)**. The statistical test used is the Friedman test with Dunn’s multiple comparisons post-test. Exact *p*-values and comparisons are indicated in the graph.

To characterize the phenotype of monocyte-derived cells acquired after the treatment with Dec-CM, we tested activation/maturation markers as HLA-DR, CD86, and CD83 expressions. We observed that monocyte-derived cells cultured with Dec-CM showed a higher expression of HLA-DR (Figures 2A,B,D) compared with culture medium. In fact, it also increased the expression of the maturation marker CD83 (Figures 2C,E). Surprisingly, Dec-CM increased the frequency of CD83^+^CD86^low^ while it diminished the frequency of the CD86^high^ population (Figures 2C,E). In line with its mature phenotype, monocyte-derived cells differentiated with Dec-CM displayed significantly lower endocytic ability in an ovalbumin (OVA)-FITC uptake assay (Figures 2F,G).

**FIGURE 3:**
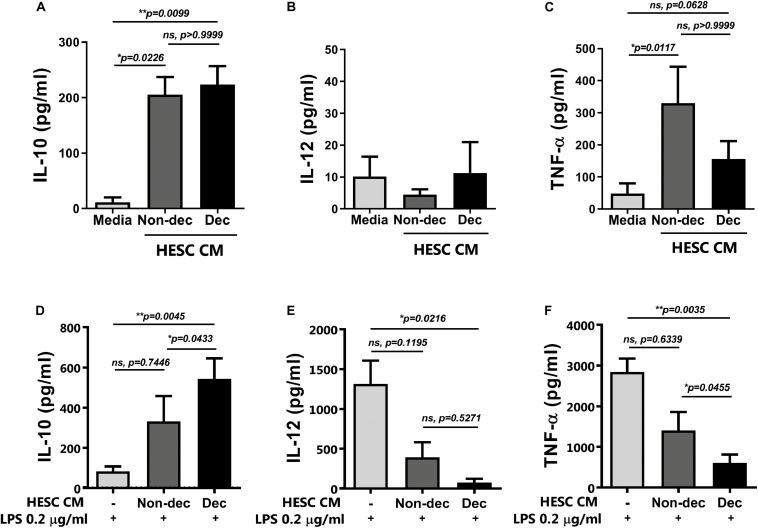
Decidualized cells induce IL-10^++^ secretion on monocyte-derived cells and prevent the increase of IL-12p70 and TNF-α secretion upon maturation with LPS. Monocytes were cultured to differentiate to immature DC in absence (Media) or presence of 1:2 dilution of Non-dec or Dec-CM for 5 days and then, cells were stimulated **(D–F)** or not **(A–C)** with LPS 0.2 μg/ml for 18 h. The secretion of IL-10, IL-12 and TNF-α was evaluated by ELISA. Bars represent the mean ± SEM of four to eight experiments. Dotted lines represent the mean of Media-treatment without LPS. The statistical test used is the Friedman test with Dunn’s multiple comparisons post-test. Exact *p*-values and comparisons are indicated in the graph.

When the cytokine secretion profile was evaluated, we observed that monocyte-derived cells differentiated in the presence of CM from HESC cells, either decidualized or not, secreted significantly higher levels of IL-10 compared to the culture medium, while IL-12 secretion was not modulated (Figures 3A,B). Notably, Dec-CM did not induce the production of TNF-α by monocyte-derived cells as Non-dec-CM did, highlighting the ability of Dec-CM to induce a different cytokine profile in these cultures (Figure 3C).

Altogether, the present results suggest that endometrial stromal cells, after decidualization, might induce an immunosuppressive regulatory phenotype on monocytes like the MRCs.

### Decidualized Cells Prevent LPS-Induced Maturation of Monocyte-Derived Cells

**FIGURE 4:**
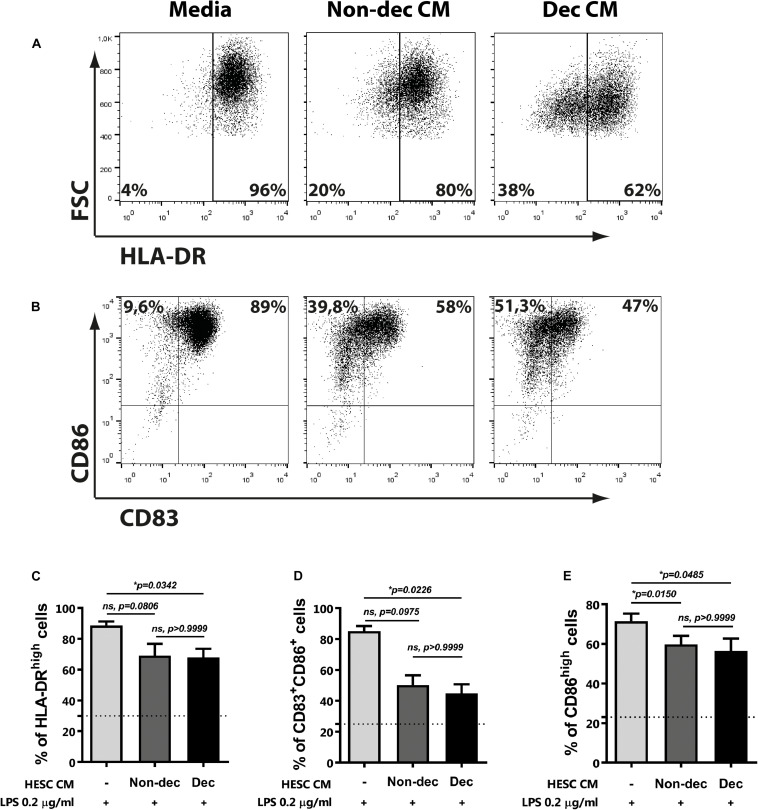
Decidualized cells prevent LPS-induced maturation of monocyte-derived cells. Monocytes were cultured to differentiate to immature DC in absence (Media) or presence of 1:2 dilution of Non-dec or Dec-CM for 5 days and then, cells were stimulated with LPS 0.2 μg/ml for 18 h. The expression of HLA-DR **(A,C)**, CD83, and CD86 **(B,D,E)** was evaluated by flow cytometry. Representative experiments are shown in panels **(A,B)** and the mean ± SEM of positive cells from five to seven experiments is shown in panels **(C–E)**. Dotted lines represent the mean of positive cells of Media-treatment without LPS. The statistical test used is the Friedman test with Dunn’s multiple comparisons post-test. Exact *p*-values and comparisons are indicated in the graph.

Next, to confirm the maturation state and the activation of monocyte-derived cells differentiated in the presence of Dec-CM, we challenged them with LPS for 16 h and cytokine profile production and the activation/maturation marker’s expression were assessed. As shown in Figure 3D, upon activation with LPS, Dec-CM cultures sustained higher IL-10 production while it prevented the increase of IL-12p70 and TNF-α secretion compared to culture medium-treated cells (Figures 3E,F). Moreover, in the presence of Dec-CM, significant increase of IL-10 and decrease of TNF-α expression in comparison with Non-dec-CM were observed, highlighting the effect of the decidualization treatment. On the other hand, the expression of activation/maturation markers in monocyte-derived cells, cultured or not, with HESC-CM and challenged with LPS was determined. Dec-CM treatment significantly prevented the increase in the frequency of HLA-DR^high^, CD83^+^CD86^+^, and CD86^high^ subsets observed with LPS treatment (Figures 4A–E). Notably, a tendency to prevent the increase in the frequency of these subsets was also observed in Non-dec-CM cultures compared to the culture medium, reaching significance in the CD86^high^ subset (Figure 4E).

The present results indicate that, once decidualized, endometrial stromal cells might not only induce a phenotype like MRCs on monocyte-derived cells but also condition their functional status.

### Decidualized Cells Favor a Higher Expression of the Characteristic Tolerogenic DC-10 Subset Markers on Myeloid Cells, HLA-G and ILT-2/CD85j

**FIGURE 5:**
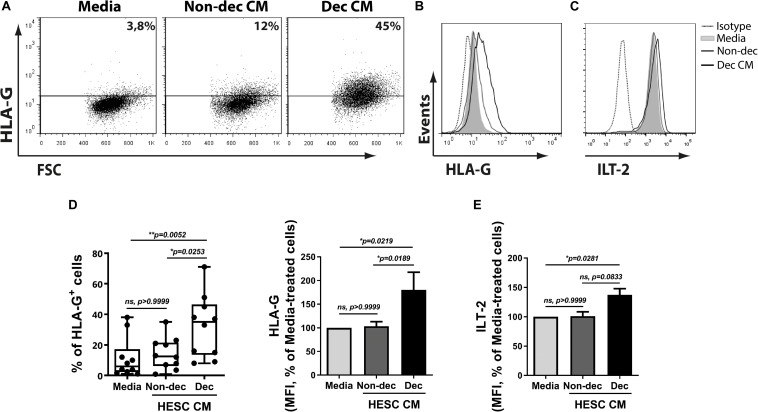
Decidualized cells favor a higher expression of the characteristic tolerogenic DC-10 subset markers on myeloid cells, HLA-G and ILT-2/CD85j. Monocytes were cultured to differentiate to immature DC in absence (Media) or presence of 1:2 dilution of Non-dec or Dec-CM for 5 days and the expression of HLA-G and ILT2/CD85j was evaluated by flow cytometry. Representative experiments are shown in panels **(A–C)** and the mean ± SEM of positive cells or MFI from six to ten experiments is shown in panels **(D,E)**. The statistical test used is the Friedman test with Dunn’s multiple comparisons post-test. Exact *p*-values and comparisons are indicated in the graph.

Based on the results shown above and considering that DC-10 spontaneously produce high amounts of IL-10 and increase tolerogenic markers, we next evaluated the ability of Dec-CM to induce tolerogenic markers on monocyte-derived cells. HLA-G expression was significantly increased in Dec-CM-treated cells compared to Non-dec-CM-treated cells. The increase of HLA-G expression was observed in both frequency and MFI parameters (Figures 5A,B,D). As expected, the expression of the HLA-G receptor, ILT-2/CD85j, was increased on monocyte-derived cells cultured in the presence of Dec-CM, compared to culture medium-treated cells (Figures 5C,E) suggesting that endometrial stromal cells might induce differentiation into the DC-10 subset compatible with a tolerogenic microenvironment only after decidualization. Interestingly, both DC-10-tolerogenic markers were not increased in those cultures treated with Non-dec-CM, highlighting the specificity of the decidualization process.

### Decidualized Cells Condition Monocyte-Derived Cells to an Immunosuppressive and Tolerogenic Profile After Allogeneic Stimulation: CD4^+^HLA-G^+^ T Cells Induction

**FIGURE 6:**
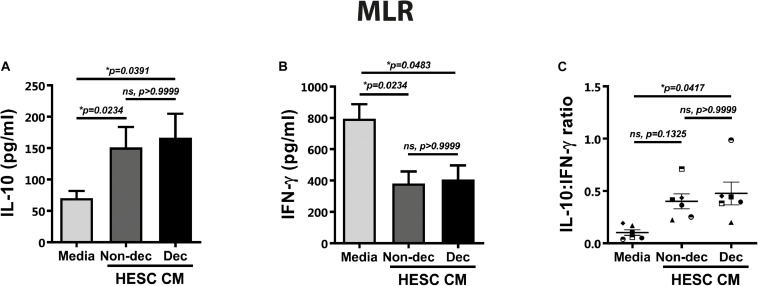
Decidualized cells condition monocyte-derived cells to an immunosuppressive profile after allogeneic stimulation. Monocyte-derived cells (5 × 10^4^ cells/100 μl) that had been differentiated in presence or absence of 1:2 dilution of Non-Dec or Dec-CM for 6 days were suspended in DC complete medium and co-cultured with allogeneic lymphocytes for 5 days more (DC/lymphocyte ratio = 1/5). On the last day of MLR, the IL-10 and IFN-γ secretion was evaluated by ELISA. Bars represent the mean ± SEM of six experiments **(A,B)** and scatter dot-plots represent the mean ± SEM of the IL-10:IFN-γ ratio production **(C)**. The statistical test used is the Friedman test with Dunn’s multiple comparisons post-test. Exact *p*-values and comparisons are indicated in the graph.

**FIGURE 7:**
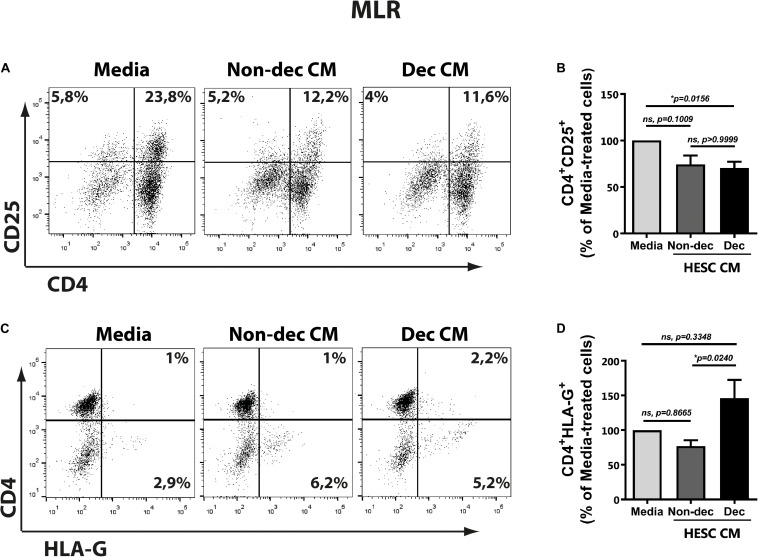
Decidualized cells condition monocyte-derived cells to an immunosuppressive and tolerogenic profile with induction of CD4^+^HLA-G^+^ T cells after allogeneic stimulation. Monocyte-derived cells (5 × 10^4^ cells/100 μl) that had been differentiated in presence or absence of 1:2 dilution of Non-Dec or Dec-CM for 6 days were suspended in DC complete medium and co-cultured with allogeneic lymphocytes for 5 days more (DC/lymphocyte ratio = 1/5). On the last day of MLR, the expression of CD4, CD25, and HLA-G was evaluated by flow cytometry, gating on lymphocytes. Representative dot plots are shown in panels **(A,C)**. Bars represent the mean ± SEM of positive cells from four experiments **(B,D)**. The statistical test used is the Friedman test with Dunn’s multiple comparisons post-test. Exact *p*-values and comparisons are indicated in the graph.

As described above, the DC-10 subset induced regulatory T cells with suppressor function through the IL-10-dependent HLA-G pathway. Taking into account the higher expression of HLA-G and ILT-2 markers in Dec-CM cultures, we evaluated the ability of these conditioned monocyte-derived cells to induce a tolerogenic and suppressor response after allogeneic stimulation in the mixed lymphocyte reaction (MLR). Hence, monocyte-derived cells that had been differentiated in the presence or absence of HESC-CM for 6 days were cultured with allogeneic lymphocytes for 5 days more. On the last day of MLR, we evaluated the expression of different markers on T cells by flow cytometry and their cytokine production profile by ELISA. An anti-inflammatory microenvironment characterized by higher IL-10 and lower IFN-γ production was observed in cultures treated with either HESC-CM, compared to medium cultures (Figures 6A,B). These results suggest the induction of suppressor and regulatory profiles on T cells in both MLR cultures, although the IL-10:IFN-γ ratio was significantly higher only in Dec-CM cultures (Figure 6C). In parallel, we evaluated the expression of the activation marker CD25, on allogeneic lymphocytes in these MLR cultures. We observed a significant decrease in the frequency of CD4^+^CD25^+^ cells in Dec-CM-cultures (Figures 7A,B), suggesting that monocyte-derived cells differentiated with CM of decidualized cells inhibited allogeneic CD4^+^ T cells activation. Finally, a significant increase in the frequency of CD4^+^HLA-G^+^ cells was observed in Dec-CM-cultures compared with Non-dec-CM-cultures, indicating a specific effect of decidualization (Figures 7C,D).

The present results suggest that HESC cells condition the monocyte-derived cells to an immunosuppressive profile accompanied by a decrease in the frequency of allo-activated T cells. Particularly Dec-CM-treated cells displayed a higher IL-10:IFN-γ ratio production and an increase in the CD4^+^HLAG^+^ T cells subset.

## Discussion

For many years, it was assumed that decidualized cells had a structural, passive role during embryo implantation, only associated with morphological changes of stromal cells. Nowadays, evidence indicates that the decidualization program conditions the endometrium for receptivity as well as for local leukocyte profiling (1, 27, 28). In fact, defects in decidualization could condition future pregnancies as observed in women with severe preeclampsia (29).

Here, we provide new experimental evidence on the decidualization program as a conditioning factor for the differentiation of maternal monocytes to a unique and special subset of Tol-DC, the DC-10, thus contributing to the establishment of tolerogenic and immune suppressor milieu by regulatory T cells induction. Our conclusions are based on several observations. First, Dec-CM inhibited monocyte differentiation to a classical CD1a^+^CD14^–^ immature DC profile in a concentration-dependent manner. Instead, CM induced a particular MRC-profile with a mature state and a higher IL-10 production on monocyte-derived cells. Moreover, Dec-CM prevented the increase of co-stimulatory molecules expression and pro-inflammatory cytokines production induced by LPS-stimulation. Finally, monocyte-derived cells differentiated in the presence of Dec-CM, expressed a higher level of DC-10-tolerogenic markers, HLA-G and ILT-2/CD85j, resulting in an immune suppressor and tolerogenic response with a higher IL-10:IFN-γ ratio and an increased frequency of regulatory CD4^+^HLAG^+^ T cells.

Stromal cells are non-hematopoietic cells; however, they have the ability to mediate anti-inflammatory effects through targeting natural killer cells, monocyte/macrophages, DCs and inducing Tregs (30–32). The mechanisms involve cell contact and the production of soluble factors, including Indoleamine 2,3-dioxygenase (IDO), TGF-β, IL-10, PGE2, and nitric oxide among other chemokines and cytokines (30–32). In this sense, the endometrium suffers regular cycles of menstruation, repair, proliferation, and differentiation under hormonal control. Endometrial leukocytes and derived-mediators play important roles not only in the decidualization and embryo implantation but also as local regulators in menstrual tissue breakdown and endometrial repair (33). It was reported that during the pre-decidualized phase the endometrium switches from a pro-inflammatory to an anti-inflammatory microenvironment (33). In this line, we observed that monocyte-derived cells differentiated in presence of Non-dec-CM also acquired some features similar to those observed in Dec-CM cultures such as HLA-DR expression, IL-12 production, and CD4^+^CD25^+^ frequency with higher IL-10 and lower IFN-γ production after MLR. Therefore, Non-dec-CM might induce an anti-inflammatory profile on DC, though it is not as marked as the one induced by Dec-CM. Thus, we might infer that DC would begin to acquire characteristics associated with an anti-inflammatory profile during the pre-decidualization phase, reaching a more robust tolerogenic profile during the decidualization process.

Here, we demonstrated that monocyte-derived cells differentiated in the presence of Dec-CM exhibit a particular CD83^+^CD86^low^ mature status with high expression of HLA-DR and spontaneous production of high amounts of IL-10. Accordingly, in the last few years, Gregori et al. characterized a subset of Tol-DC, the DC-10 subset, which can be differentiated *in vitro* from monocytes with GM-CSF + IL-4 and IL-10 (11). DC-10 are CD1a^–^CD14^+^ and display a mature myeloid phenotype (CD83^+^CD86^+^ and HLA-DR^high^) even in the absence of activation stimuli. Moreover, they secrete spontaneously high levels of IL-10 and express the tolerogenic markers HLA-G, ILT-2, ILT-3, and ILT-4. This phenotype turns them into potent inducers of Tr1 *in vitro* through the IL-10-dependent ILT-4/HLA-G pathway (11).

Although DC-10 share some similarities with other tolerogenic antigen-presenting cells, they represent a unique subset of Tol-DC that is phenotypically and functionally stable (34). Upon activation, DC-10 maintain their phenotype and their cytokine secretion profile with high IL-10 and low IL-12/TNF-α production. In accordance, here we showed that Dec-CM prevented the maturation of DC by LPS, inhibited IL-12 and TNF-α production, and increased even more the secretion of IL-10. The presence of DC-10 was recently reported in first trimester decidua and peripheral blood (6). However, it is still unclear if the increased frequency of DC-10 observed in the decidua is due to: (a) a higher recruitment from peripheral blood, (b) an increased conversion of resident decidual DCs into DC-10, or (c) if the decidual microenvironment promotes the *de novo* induction of DC-10 from monocytes recruited. Regarding the aforementioned frequency and based on the results presented here, we propose that *the novo* induction of DC-10 could be occurring within human decidua and independently of the blastocyst presence.

The frequency of DCs in human endometrium reaches its highest level during the implantation window (35) and it is associated to their ability to release soluble factors that improve the endometrial receptivity (36, 37). In this context, previous work performed in mice provided strong evidence of the indispensability of DCs in decidua formation and implantation (38, 39). IL-10 promotes the expression of several tolerogenic molecules in human DCs and in other antigen-presenting cells, including IL-10 itself, hemo-oxygenase (HO-1), ILT-3, and ILT-4 as well as another important mediator of immune tolerance in pregnancy, the HLA-G. This atypical MHC class I molecule, is one of the ILT-2/ILT-4 ligands with potent immunosuppressive properties (40).

In accordance with these observations, in our *in vitro* model of immune-decidual interaction, we demonstrated that Dec-CM was able to induce HLA-G and increased ILT-2 expression on monocyte-derived cells. Interestingly, these DC-10-tolerogenic markers were not increased at all by Non-dec-CM treatment. It has been reported that the continuous ligation of ILT-2 on immature DC during differentiation maintains CD14 expression, inhibits the acquisition of CD1a expression and prevents the activation with LPS (41, 42). In fact, ligation through ILT-2 may preferentially induce and/or interact with Tregs that maintain T-cell unresponsiveness (42). Considering that the decidualization process increased IL-10 production by stromal cells (43), which induces the expression of IL-10, HLA-G and ILT-4 on DC-10 (11), and that HLA-G itself is able to up-regulate the expression of ILT-2 and ILT-4 (44), we suggest that Dec-CM induces a positive regulatory loop between IL-10 and these tolerogenic markers in monocyte-derived cultures.

*In vitro* experiments have demonstrated that DCs isolated from the decidua (45) are poor stimulators of allogeneic lymphocytes. Accordingly, here we showed for the first time that allogeneic lymphocytes co-cultured with monocytes-derived cells conditioned by Dec-CM treatment are hypo-responsive with significantly decreased CD25^+^ expression, particularly on the CD4^+^ subset. Indeed, a significantly higher IL-10/IFN-γ ratio was observed in these cultures, suggesting the induction of a regulatory T-cell profile. In this sense, new subsets of regulatory T cells have emerged, defined by the expression of the HLA-G cell surface; CD4^+^ and CD8^+^ HLA-G^+^ T cells. They were identified in peripheral blood of healthy volunteers as small subsets but were able to suppress immune responses *in vitro* involving IL-10 and HLA-G as suppressive mechanisms (46–48). According to our results, high IL-10 and low IFN-γ secretion mediated by CD4^+^HLA-G^+^ T cells was previously reported by other authors and, therefore, the ability of this small regulatory T cell subset to promotion of an anti-inflammatory or antiproliferative cytokine milieu has been suggested (46–48). Here, we showed that Dec-CM induced the differentiation of regulatory HLA-G^+^ T cells in monocytes-derived cell cultures while Non-dec CM was unable to induce this particular subset of regulatory CD4^+^ T cells, highlighting characteristic properties of the decidualization process. It was demonstrated that CD4^+^ T cells might acquire the HLA-G molecule from decidual DCs through the trogocytosis process (18). In fact, it was proposed that DC-10-derived extracellular vesicles also contain soluble HLA-G (sHLA-G) and T cells can acquire HLA-G (49). Although we demonstrated the presence of CD4^+^HLA-G^+^ T cells on MLR cultures performed with total lymphocytes, we observed a low frequency of this subpopulation, suggesting that their physiological relevance would be based on suppressive capacity through the production of high levels of IL-10 and sHLA-G. However, more functional studies should be performed to address this issue.

Here we also observed an increase in the non-CD4^+^HLA^–+^ cells after the MLR cultures. In this sense, a higher frequency of CD8^+^HLA-G^+^ T cells cells in the peripheral blood of healthy pregnant compared to non-pregnant women, was recently reported (50). Taking into account that there are few studies on HLA-G^+^ T cell subsets in the context of pregnancy, and are even less focused on CD8^+^HLA-G^+^, it would be interesting to perform functional studies to characterize this unexplored regulatory subset (6, 18, 50).

Considering the present results, we suggest that decidual regulatory HLA-G^+^ T cells could be induced locally by DC-10 which were previously differentiated in the pre-implantation period by soluble factors released by decidualized cells. However, we cannot exclude that the HLA-G^+^ T cells could also be recruited toward the decidua from the periphery. Finally, even though we demonstrated that, through soluble factors, the decidualized cells induce DC-10 and condition the T cell profile toward a tolerogenic one by the induction of regulatory T cells, it still remains to be defined whether these mechanisms operate in the human decidua *in vivo* and how they cooperate in promoting and maintaining feto-maternal tolerance.

## Data Availability Statement

All datasets generated for this study are included in the article.

## Ethics Statement

The studies involving human participants were reviewed and approved by Investigation and Ethics Committee from “Academia Nacional de Medicina.” The participants provided their written informed consent to participate in this study.

## Author Contributions

GS, CP, and RR designed the study, supervised the experimental work, and wrote the manuscript. SG carried out all the experiments using dendritic cells. ES, LF, and EG performed the HESC cells treatments. SG, EG, LG, FM, and LF did the flow cytometry analysis, ELISA assays, and interpretation analysis. SG, AC, and MB did the purification of monocytes and lymphocytes used in this work. GS and RR supervised the whole study. All authors read and approved the final manuscript.

## Conflict of Interest

The authors declare that the research was conducted in the absence of any commercial or financial relationships that could be construed as a potential conflict of interest.
